# The Effects of Different Treatments on Serum Trace Element Levels in Dogs with Heart Failure

**DOI:** 10.3390/ani14233390

**Published:** 2024-11-25

**Authors:** Bengü Bilgiç, Duygu Tarhan, Mehmet Erman Or

**Affiliations:** 1 Department of Internal Medicine, Faculty of Veterinary Medicine, Istanbul University-Cerrahpasa, Istanbul 34320, Turkey; ermanor@iuc.edu.tr; 2 Department of Biophysics, School of Medicine, Bahcesehir University, Goztepe, Istanbul 34734, Turkey; duygu.tarhan@bau.edu.tr

**Keywords:** dogs, heart failure, trace elements

## Abstract

The prognostic and diagnostic role of some trace elements in heart diseases has been demonstrated. In this study, the effects of min. 30-day and min. 120-day treatment with pimobendan, enalapril, and furosemide, as monotherapy and in combination, on serum trace element levels in dogs with heart failure were evaluated. A total of 107 dogs were treated with pimobendan or enalapril as monotherapy and pimobendan + enalapril (PE), enalapril + furosemide (EF), or pimobendan + enalapril + furosemide (PEF) as combination therapy for a min. of 30 and 120 days. Serum copper (Cu), zinc (Zn), iron (Fe), cobalt (Co), magnesium (Mg), manganese (Mn), selenium (Se), and chromium (Cr) were measured by an inductive coupled plasma optical emission spectroscopy device. Mean serum Mg in dogs treated with pimobendan for a min. of 120 days was significantly lower than that for a min. of 30 days (*p* < 0.05). In dogs using the PEF combination, mean serum Fe was significantly higher in the min. 120-day treatment group than in the min. 30-day treatment group (*p* < 0.01). No significant difference was observed in mean serum Cu, Zn, Co, Mn, Se, or Cr between the treatment groups and the time periods (*p* > 0.05). The short- and long-term use of pimobendan, enalapril, furosemide, and their combinations may cause changes in mean serum Mg and Fe in dogs with heart failure.

## Simple Summary

Some drugs may affect the absorption and excretion of trace elements and cause changes in their serum levels. In this study, the effects of short- (min. 30 days) and long-term (min. 120 days) treatment with pimobendan, enalapril, and furosemide, as monotherapy and in combination, on serum copper, zinc, iron, cobalt, magnesium, manganese, selenium, and chromium levels in dogs with heart failure were evaluated. Our findings suggest that the short- and long-term use of pimobendan, enalapril, furosemide, and their combinations may cause changes in mean serum magnesium and iron element levels in dogs with heart failure.

## 1. Introduction

Various cardiac, hemodynamic, renal, neurohormonal, and cytokine-related abnormalities may affect the progression of valvular lesions in dogs. The American College of Veterinary Internal Medicine (ACVIM) reported an updated consensus statement in 2019 for minimizing the risks, classification, and monitoring of disease and also appropriate diagnosis and treatment approaches. Based on the severity or stage of myxomatous mitral valve disease, pharmacological or dietary interventions are recommended by the ACVIM. Commonly prescribed medications for managing heart disease in affected dogs include pimobendan, enalapril, and furosemide, which may be used as monotherapy or in combination therapy depending on the clinical condition of the patient [1].

Pimobendan is a benzimidazole-pyridazinone derivative approved for use in the treatment of congestive heart failure (CHF) secondary to chronic myxomatous mitral valve disease (MMVD) and dilated cardiomyopathy (DCM) in dogs. As a Ca^2+^ sensitizer and phosphodiesterase 3 (PDE3) inhibitor, it has vasodilator and inotropic effects on the heart. Pimobendan’s active metabolite, UD-CG 212 Cl, acts as a competitive antagonist for A1-adenosine receptors. Blocking the inhibitory effects of A1-adenosine receptor stimulation on the heart causes cyclic adenosine monophosphate (cAMP) synthesis and positive inotropy [2]. Enalapril exhibits antihypertensive effects due to its active metabolite, enalaprilat, which inhibits the angiotensin-converting enzyme (ACE). This enzyme converts angiotensin I, an inactive precursor, into angiotensin II, a potent vasoconstrictor [3]. It also stimulates venous and arterial vasodilation and increases peripheral venous capacitance, reducing right atrial, pulmonary artery, and capillary vessel pressures and left ventricular filling volume in dogs with congestive heart failure (CHF) [4]. Loop diuretics are commonly used for the treatment of CHF characterized by Na^+^ and water retention in humans and animals due to their significant effects such as lowering intravascular hydrostatic pressure and reducing the clinical symptoms associated with edema [5].

Trace elements are essential for the optimal maintenance of cardiac functions. There are many studies investigating the relationships between abnormal trace element concentrations and cardiovascular diseases [6,7,8,9]. Experimental studies have shown some interactions between glucose intolerance, hypercholesterolemia, abnormal electrocardiography (ECG), hypertension, and inadequate copper (Cu)-containing diet intake [10,11]. In a study evaluating the correlation between various cardiac biomarkers and elements in human medicine, iron (Fe) was significantly positively correlated with cardiac troponin-I (cTnI) and both zinc (Zn) and selenium (Se) were significantly negatively correlated with cardiac troponin-T (cTnT), which suggests that these correlations may have positive prognostic value in patients with acute coronary syndrome [12]. In patients with ischemic heart disease, Cu affects myocardial contractility, and magnesium (Mg), calcium (Ca), manganese (Mn), Cu, and Zn are associated with cardiac arrhythmias [13]. It has been reported that Mg deficiency may be associated with cardiovascular diseases such as ischemic heart disease, CHF, sudden cardiac death, atherosclerosis, and cardiac arrhythmias [14,15,16,17,18]. Studies investigating the relationship between Zn deficiency and cardiovascular diseases in human medicine have reported that low Zn levels may be a risk factor in the formation of atherosclerosis, coronary artery disease, ischemic heart disease, hypertension, myocardial infarction, atrial fibrillation, and CHF [19,20]. Many studies have reported abnormal ECG findings with blood pressure changes in mice and sheep [21,22], epicardial and myocardial hemorrhages in pigs [23], and degenerative skeletal muscle disorders in poultry [24] fed with a low Se-containing diet.

Studies investigating the effects of medication on serum trace element levels in veterinary medicine are quite limited in the literature. This study aimed to determine the effects of pimobendan, enalapril, and furosemide as monotherapy and in combination on serum trace element levels in short and long treatment periods and to evaluate the indication of element supplementation during the management of disease.

## 2. Materials and Methods

### 2.1. Animals and Treatment Groups

A total of 107 dogs of various breeds, ages, weights, and sexes were used, and the ethics committee’s approval of the experimental study was received from the local ethics committee of Istanbul University-Cerrahpasa (Ethics Committee No: 2020/03). The study population included 68 male and 39 female dogs of various breeds, such as Yorkshire terrier (*n* = 6), Labrador retriever (*n* = 1), mixed breed (*n* = 12), English cocker spaniel (*n* = 11), golden retriever (*n* = 7), Cavalier King Charles spaniel (*n* = 39), pug (*n* = 2), Pomeranian (*n* = 3), pincher (*n* = 8), Maltese terrier (*n* = 4), Chihuahua (*n* = 3), beagle (*n* = 1), Pekingese (*n* = 6), German shepherd (*n* = 1), and Shih Tzu (*n* = 3). The minimum and maximum age and body weight were 4–18 years and 3–43 kg, respectively. All the dogs were owned, kept at home, and fed similar commercial foods throughout the study. Considering the ACVIM consensus statement, dogs were administered short- and long-term drug therapy according to the findings of the general and cardiopulmonary examinations (Table 1). Dogs diagnosed with heart disease were grouped as pimobendan (P) (*n* = 20) at a dosage of 0.25–0.3 mg/kg PO q12h and enalapril (E) (*n* = 25) at a dosage of 0.25–0.5 mg/kg PO q12h as monotherapy and pimobendan and enalapril (PE) (*n* = 22), enalapril and furosemide (EF) (*n* = 20), and pimobendan, enalapril, and furosemide (PEF) (*n* = 20) as combination therapy. All the treatment groups were subdivided into a minimum of 30 days (*n* = 12, 8, 12, 14, and 9 for P, E, PE, EF, and PEF groups, respectively) and a minimum of 120 days (*n* = 8, 17, 10, 6, and 11 for P, E, PE, EF, and PEF groups, respectively) according to the treatment periods. Furosemide at a dosage of 2–8 mg/kg was administered.

Dogs diagnosed with both asymptomatic (B2) and symptomatic (C and D) stages of MMVD according to the ACVIM classification (*n* = 96), tricuspid valve disease (*n* = 3), DCM (*n* = 6), and congenital heart diseases (*n* = 2) were included in the study.

### 2.2. Inclusion/Exclusion Criteria

The inclusion criteria were as follows: hemogram and biochemistry results within the reference ranges, being fed with stable dry pet food, and being kept indoors. The exclusion criteria were as follows: a diagnosis of single or multiple organ failure, infectious, metabolic, or systemic diseases concurrent with heart failure; indications of different systemic medications or vitamin or mineral supplementation; indications of treatment with digoxin, dobutamine, nitroglycerin, nitroprusside, spironolactone, beta blockers, or bronchodilators concurrent with the treatment protocols in this study.

### 2.3. Clinical and Cardiovascular Examinations

Vital signs such as body temperature, capillary refilling time, respiratory and pulse rate and quality, mucosal membranes, hydration status, systolic, diastolic, mean arterial pressures, and auscultation findings were recorded in all patients between 2019 and 2022. Blood pressure was measured by a BioCare^®^ Pettrust device (BioCare, Taoyuan City, Taiwan). The vertebral heart scale (VHS) was measured on laterolateral (LL) thorax radiographs taken and evaluated for cardiomegaly. Electrocardiographic examinations were performed by ECG600G device (CONTAC^®^, Qinhuangdao, China) and echocardiographic examinations were performed by Apogee 3500 V (SIUI^®^, Shantou, China) and Vetus 8 Doppler devices (Mindray^®^, Shenzhen, China). Right parasternal long- and short-axis and left apical four-chamber images were recorded. The hemogram and biochemistry analyses of all patients were evaluated.

### 2.4. Trace Element Measurements

The collected blood serums were gradually stored at room temperature for the first 3–4 h and then at −20 °C for 2 days and at −80 °C until the measurement day. Cu, Zn, Fe, Co, Mg, Mn, Se, and Cr were measured by an inductively coupled plasma optical emission spectroscopy, iCAP 6000 series (ICP-OES) device (Thermo Fisher Scientific^®^, Waltham, MA, USA). The ICP-OES device parameters used in the trace element measurements are presented in Table 2. For all trace element measurements, appropriate test solutions containing 2000 ppm (mg/L) for each element, obtained from Chem-Lab NV (Thermos Fisher Scientific, Cambridge, UK), were used for the quality assurance of the ICP-OES device. Standard solutions of all elements were prepared using solutions in deionized water containing 1000 ppm (mg/L) for each element obtained from Chem-Lab NV (Certified reference material; Belgium; Cu solution lot: 18.2051801.30, Zn solution lot: 18.0280206.50, Fe solution lot: 17.1491611.20, Co solution lot: 18.2552307.20, Mg solution lot: 18.1361301.50, Mn solution lot: 18.0160206.50, Se solution lot: 18.2031805.20, and Cr solution lot: 19.1021101.5). Measurements were performed after using these standard solutions and deionized water as a blank solution. Reproducible and linear calibration curves were obtained by using standard and blank solutions. Serum samples were diluted 1:10, with distilled water used as a blank solution. Thus, the correlation coefficient of the calibration curve was found for each of the elements measured. The recovery of the analyzed quality control was between 92.8% and 107.6%. Table 3 shows the results of the ICP-OES method validation. In the study, the appropriate wavelengths, as given in Table 4, of all elements were used for the analysis using the ICP-OES device. Measurements of all samples were performed on the same day and with the same calibration to avoid being affected by weather conditions such as temperature and humidity or by device calibration. The results were expressed as mg/L (ppm).

### 2.5. Statistical Analysis

To compare the treatment duration of a min. of 30 and 120 days, the Mann–Whitney U test was used for all elements except the normally distributed Se. To compare the different drugs in each treatment duration subgroup, the Kruskal–Wallis test was applied for all elements except Se. The independent samples *t*-test was used to compare treatment durations, and one-way analysis of variance (ANOVA) was used to compare different treatment groups for Se values. Data were expressed as mean ± standard deviation (SD). All statistical analyses were performed using the SPSS 25.0 program, and *p* < 0.05 was considered statistically significant.

## 3. Results

**Figure 1:**
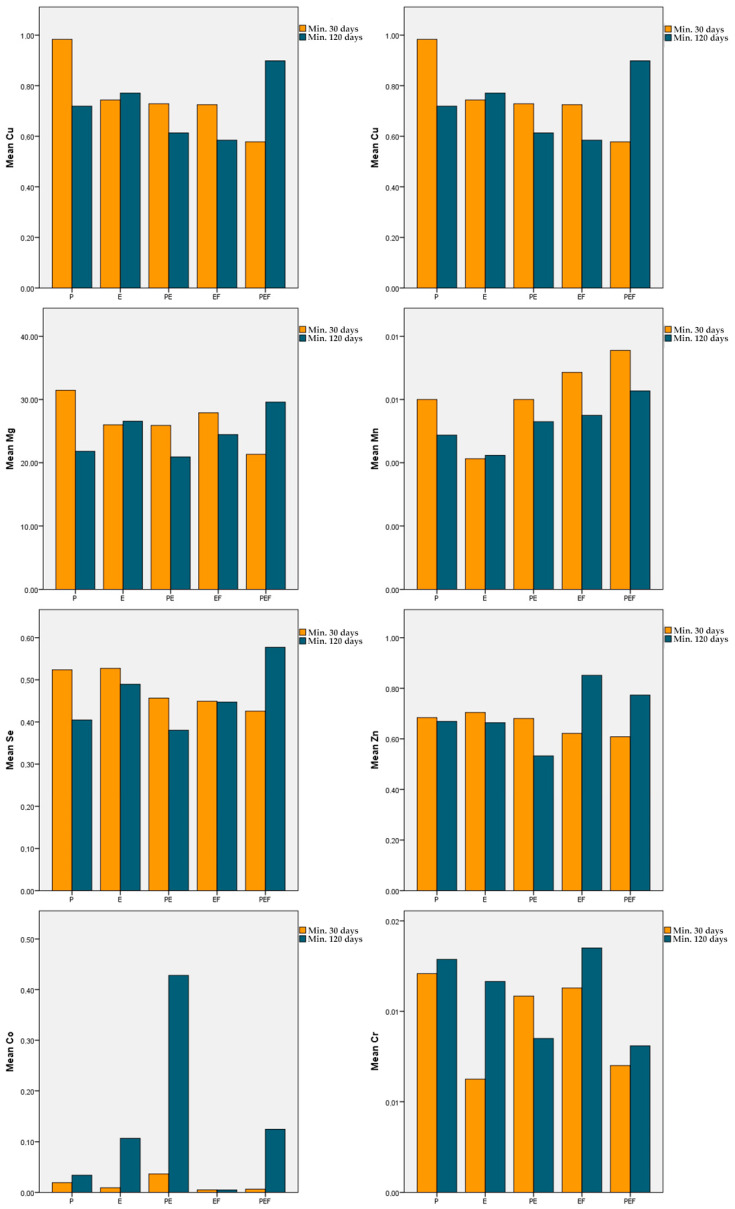
Mean serum Cu, Zn, Fe, Co, Mg, Mn, Fe, and Cr in each treatment group.

The mean serum Cu level in P, PE, and EF was lower and in E and PEF was higher in the min. 120-day treatment duration group than in the min. 30-day group. However, there was no significant difference in the mean serum Cu level between all study groups (*p* > 0.05). In the comparison of treatment groups within both treatment durations, no significant difference was observed (*p* > 0.05) (Table 5) (Figure 1).

The mean serum Zn level in P, E, and PE was lower and in EF and PEF was higher in the min. 120-day treatment duration group than in the min. 30-day group. However, there was no significant difference in the serum Cu level between all study groups (*p* > 0.05). In the comparison of treatment groups within both treatment durations, no significant difference was observed (*p* > 0.05) (Table 6) (Figure 1).

The mean serum Fe value in P and PE was lower and in E and EF was higher in the min. 120-day treatment duration group than in the min. 30-day group. However, these differences were not statistically significant (*p* > 0.05). The mean serum Fe level in PEF increased significantly in the min. 120-day group compared with the min. 30-day group (*p* < 0.01). In the comparison of treatment groups within the 120-day treatment duration, the mean serum Fe level was significantly lower in PE than in E and PEF (*p* < 0.05) (Table 7) (Figure 1).

The mean serum Co value in P, E, PE, and PEF was higher in the min. 120-day treatment group than in the min. 30-day group. However, these differences were not statistically significant (*p* > 0.05). In EF, the mean serum Co level was measured at the same concentration in both treatment duration groups. In the comparison of treatment groups within both treatment durations, no significant difference was observed (*p* > 0.05) (Table 8) (Figure 1).

The mean serum Mg levels in E and PEF were higher and in PE and EF were lower in the min. 120-day treatment duration group than in the min. 30-day group. However, these differences were not statistically significant (*p* > 0.05). The mean serum Mg level in P was significantly decreased in the min. 120-day treatment duration group compared to the min. 30-day group (*p* < 0.05). In the comparison of treatment groups within the min. 30-day treatment duration, the mean serum Mg level of PEF was significantly lower than that of E (*p* < 0.05) (Table 9) (Figure 1).

The mean serum Mn levels in P, PE, EF, and PEF were lower in the min. 120-day treatment duration group than in the min. 30-day group. However, these differences were not statistically significant (*p* > 0.05). In addition, it was observed that the mean serum Mn level in E was measured at the same concentration in the short- and long-term treatment groups (*p* > 0.05). In the comparison of treatment groups within both treatment durations, no significant difference was observed (*p* > 0.05) (Table 10) (Figure 1).

The mean serum Se level in P, E PE, and EF was lower and in PEF was higher in the min. 120-day treatment duration group than in the min. 30-day group. However, these differences were not statistically significant (*p* > 0.05). In the comparison of different treatment groups, no significant difference was observed within either treatment duration (*p* > 0.05) (Table 11) (Figure 1).

The mean serum Cr value in E, EF, and PEF was higher and in PE was lower in the min. 120-day treatment duration group than in the min. 30-day group. However, these differences were not statistically significant (*p* > 0.05). In addition, it was observed that mean serum Cr levels in P were at the same concentration in both the short- and long-term treatment duration groups (*p* > 0.05). In the comparison of different treatment groups, no significant difference was observed within either treatment duration (*p* > 0.05) (Table 12) (Figure 1).

## 4. Discussion

Active substances of certain drugs may affect the absorption, excretion, and serum concentrations of trace elements [25,26,27,28,29,30]. Moreover, various systemic diseases may cause dramatic changes in serum element concentrations [31,32]. In a previous study, serum trace element levels were investigated in dogs diagnosed with MMVD, and no significant difference was observed in patients compared to healthy controls [33]. Considering the results of this study, we comparatively investigated the effects of the drugs commonly used in veterinary cardiology on similar trace elements.

Cu deficiency has been demonstrated in various studies to contribute to the development of cardiovascular disorders, including myocardial infarction, papillary muscle rupture, vascular lesions, cardiomegaly, cardiac arrhythmias, cardiac hypertrophy [34,35], ischemic cardiac damage [36,37], infarction [38], cardiomyopathies [39], cardiac defects [40,41], and CHF [41]. Considering these studies examined the relationship between cardiovascular diseases and Cu, the importance of optimum Cu concentrations for cardiovascular health in dogs may be seen. One of the major factors affecting Cu levels in living organisms is some systemic drugs used short or long term. Various drugs, such as D-penicillamine, trientine, and Zn salts, reduce the absorption or increase the excretion of Cu levels [42]. In human medicine, the Cu binding activity of the anticarcinogenic agents disulfiram, clioquinol, and diethyldithiocarbamate has been reported [25,26]. In a study conducted in patients with DCM, treatment with diuretics did not change the mean serum Cu levels significantly [43]. According to our results, enalapril, pimobendan, and furosemide, both as monotherapy and in combination, did not have a significant role in Cu absorption and excretion. Although long-term treatment with diuretics is known to induce the excretion or retention of some macro-elements [44,45], our findings are consistent with the results of Cunha et al.

In human medicine, it has been reported that ACE inhibitors and diuretics increase urinary Zn excretion and may predispose to Zn deficiency [27,28]. Wester et al. [46] reported that thiazide and loop diuretics increased urinary Zn excretion but did not cause a significant difference in the mean serum Zn level after treatment. In a study evaluating the effects of diuretics, beta blockers, Ca antagonists, ACE inhibitors, and ACE II receptor antagonists on serum Zn levels, a significant decrease in the mean serum Zn concentration was noted in patients treated with diuretics [29]. Additionally, diuretics such as thiazide and chlorthalidone, which act mainly in the first part of the distal convoluted tubule, have been shown to significantly increase the urinary excretion of Zn [47]. Unlike previous studies in human medicine that reported a decrease in serum Zn concentrations related to diuretics and ACE inhibitors [27,28,47], enalapril and furosemide did not affect serum Zn levels in our study. In a study on rats, the effects of oral furosemide and spironolactone treatment on urine and serum Zn levels were evaluated, and it was reported that short-term treatment with both drugs increased the urinary excretion of Zn and also increased the serum Zn levels compared to the control group [48]. As a result, it was suggested that since the Zn concentration in the blood can be increased by various compensatory mechanisms, such as increasing absorption from the gastrointestinal tract or using Zn stores in case of Zn deficiency, measuring Zn levels is not a reliable method to evaluate the Zn status in living organisms [49]. In a previous study, although an increase in urinary Zn excretion was reported in patients with hypertension treated with captopril, no significant changes in serum Zn levels were observed in either the captopril or enalapril groups [28]. In another similar study conducted by O’Connor et al. [50], it was reported that captopril treatment in patients with hypertension did not cause a significant change in serum Zn levels. These results are consistent with our study.

Although the relationship between heart failure and serum Fe levels has been explained by various mechanisms in human medicine, studies are quite limited in veterinary medicine. In a retrospective study, the prevalence of Fe deficiency in dogs with heart disease was reported as 18% [51]. In human medicine, it was suggested that although in the early stages of heart failure, anemia does not occur due to increased hepcidin levels, in the advanced stages of the disease, Fe deficiency and anemia may develop as the circulating hepcidin level decreases [52]. Hepcidin, a peptide primarily synthesized and secreted by hepatocytes, is encoded by the HAMP gene located on chromosome 19. It serves as the principal regulator of systemic iron homeostasis, governing both iron absorption and release [53]. Hepcidin regulates the activity of ferroportin, a transmembrane protein responsible for exporting iron from various cell types, including duodenal gut mucosal cells at the site of iron absorption and hepatocytes and macrophages at the site of iron storage. Upon binding to ferroportin, hepcidin triggers its degradation in lysosomes, thereby reducing iron release into circulation [54]. In patients with heart failure, iron intake is often reduced, and absorption may be impaired due to various mechanisms, including diminished gastric acidification, gut wall edema, and disrupted ferroportin activity. Enhanced inflammation, characterized by increased hepcidin expression, plays a key role in suppressing ferroportin function. Additionally, volume expansion, commonly observed during acute decompensations of heart failure, may contribute to further reductions in plasma ferritin levels and the development of pseudo-anemia [55]. Based on these studies, a decrease in mean serum Fe levels in dogs with advanced heart disease (C and D stages in MMVD) may be expected. However, the significant increase in mean serum Fe levels in the PEF treatment group after long-term treatment does not seem to support the hepcidin mechanism in dogs with heart failure. In addition, considering the wide Fe concentration reference range in healthy dogs, the significant changes we identified in the study were within the reference ranges for dogs. Furthermore, our results revealed that furosemide did not affect serum Fe concentrations in either long- or short-term use in combination. In a study investigating liver, lung, heart, and kidney tissues and serum Fe concentrations in rats treated with furosemide, the highest Fe loss was in serum [56]. However, studies investigating the effects of furosemide on serum Fe concentrations are quite limited. Therefore, further studies on larger study groups are needed.

It is known that a high concentration of Co has toxic effects on the cardiovascular system [57]. Cobalt-induced cardiac toxicity was first identified in the early 1960s as “Quebec beer-drinkers’ cardiomyopathy”, attributed to the use of a cobalt-based foam-stabilizing agent [58]. Affected patients may present severe biventricular heart failure, notable for its sudden onset, associated pericardial effusion, low-voltage electrocardiogram findings without arrhythmias, and rapid progression to cardiogenic shock. The condition is associated with a high mortality rate of 10–40% [59]. It has been shown that NSAID and quinolone antibiotics form complexes with many elements [60]. Studies investigating serum Co–drug interactions are quite limited in the literature. In our study, we observed that long- and short-term treatment with pimobendan, enalapril, and furosemide, both as monotherapy and in combination, did not cause significant changes in serum Co levels in dogs.

There are many studies in human medicine investigating the mechanism of various drug–Mg interactions. It has been reported that cardiac glycosides such as digoxin increase renal Mg excretion and reduce Mg reabsorption in renal tubules [61,62]. Also, various thiazide and loop diuretics such as furosemide increase renal Mg loss by reducing paracellular Mg reabsorption in the ascending limb of the loop of Henle [30]. In a study conducted by Cohen et al. [63], hypomagnesemia was reported in 12.3% and hypermagnesemia was reported in 4.9% of patients with CHF treated with furosemide. Spasov et al. [64] observed that the intraperitoneal administration of 30 mg/kg of 1% furosemide solution caused moderate Mg deficiency in mice. In a study evaluating the effects of enalapril and spironolactone on serum Mg levels in dogs with DCM, it was reported that the combination of both drugs caused a significant increase in serum Mg levels over time; however, this increase was not considered clinically significant [65]. In another study investigating the effects of long-term furosemide use on serum Mg levels in rats, it was reported that furosemide treatment did not change serum Mg values, and it was suggested that chronic furosemide treatment increases Mg reabsorption in distal tubules and compensates the reduction of Mg+ 2 absorption in the ascending limb of the loop of Henle [66]. Our results are consistent with these studies. We observed that the mean serum Mg value in dogs using pimobendan as monotherapy for a min. of 120 days decreased significantly compared to patients treated for a min. of 30 days (*p* < 0.05). However, there are no clinical studies in the literature investigating pimobendan–Mg interactions. Therefore, the underlying mechanism is not clear, and further studies are needed to support these results.

Considering the toxic effects of high levels of Mn on the cardiovascular system, the appropriate concentration of Mn in dogs with heart failure is important. However, studies investigating Mn–drug interactions are quite limited. Prolonged exposure to Mn has been linked to impaired myocardial contractility. At high exposure concentrations (1–8 mmol/L), Mn not only suppresses myocardial contraction but also reduces the action potential duration, alters the effective refractory period (ERP), and diminishes the maximum upstroke velocity (Vmax) of the action potential [67]. The high-dose intravenous administration of Mn has been associated with a reduction in heart rate and blood pressure, as well as an increase in PR and QRS interval durations in the canine heart [68]. Mn exposure-related effects, such as impaired myocardial contraction, blood vessel dilation, and hypotension, indicate a substantial impact on cardiac function [69]. As a result of this study, it was observed that the short- and long-term use of pimobendan, enalapril, and furosemide did not have a significant effect on serum Mn levels.

Se acts as an antioxidant element in many tissues and organs, including the cardiovascular system. In a study investigating the effects of various diuretics and their combinations on serum Se levels in human medicine, patients with hypertension were treated with piretanide, hydrochlorothiazide, and amiloride for up to 12 weeks, and no significant change was observed in mean serum Se levels in any of the treatment groups. As a result of the study, it was stated that these drugs did not affect Se homeostasis [70]. Similarly, in a study conducted in people with idiopathic DCM using diuretics [43], mean serum Se levels in treated patients were not significantly different from those of healthy control groups. Our results are consistent with these two studies.

Previous studies have shown that some active substances of drugs may change the absorption and excretion of Cr [71,72,73]. It was suggested that phytates reduce Cr absorption, and ascorbic acid increases Cr absorption significantly [74]. In a study investigating the effects of antihypertensive drugs on Cr levels in the liver and kidney tissue of rats [29], the liver and kidney Cr concentrations in the treated group were significantly higher than those in healthy controls. As a result of the study, it was stated that the administration of amlodipine may lead to Cr accumulation in internal organs. However, in veterinary medicine, studies evaluating the effects of enalapril, pimobendan, furosemide, and their combinations are quite limited in the literature.

In order to minimize the diet-related differences of serum element levels, we limited the study to animals fed dry non-prescription food only. It is well known that diet-related element differences may occur. However, the element and mineral contents of dry foods are standardized by international regulations in order to maintain nutritionally well-balanced food production. In Europe, the European Pet Food Industry Federation (FEDIAF) has recommended guidelines related to nutrient levels for complete dog foods [75]. Accordingly, since all the dogs in the study groups were fed with complete dry dog foods throughout their lives, significant food-related changes were minimized.

MMVD is a degenerative and genetic-based disease. Similarly, mutations in several genes result in DCM in dogs [76]. MMVD and DCM are not primarily inflammatory conditions. Also, any other inflammatory conditions were excluded based on clinical examination and laboratory tests. Accordingly, inflammatory-related serum element changes were minimized in the study.

In the study, the inclusion of different diseases was a limitation. Although the subjects were fed a similar type of dry food, variations in diet and differing environmental living conditions may represent crucial limitations. The study emphasizes the importance of trace elements in cardiac health. Incorporating a trace element evaluation into the management of dogs with heart disease could lead to better outcomes by optimizing both drug efficacy and nutritional support. Monitoring possible changes in trace elements during short- and long-term treatments provides critical insights for managing chronic conditions. This can guide veterinarians in scheduling periodic evaluations and adjusting treatments accordingly.

## 5. Conclusions

In conclusion, mean serum Mg concentrations in long-term treatment with pimobendan in dogs were significantly lower than those in short-term treatment. A significant increase was observed in mean serum Fe concentrations in dogs receiving a pimobendan + enalapril + furosemide combination for a min. of 120 days compared to a min. of 30 days. However, since wide Fe reference ranges in dogs exist, the results may not seem clinically significant. In conclusion, long- or short-term treatment with pimobendan, enalapril, and furosemide, as monotherapy or in combination, has no significant effect on serum Cu, Zn, Co, Mn, Se, and Cr levels in dogs with heart disease.

## Figures and Tables

**Table 1:** Drug selection criteria based on the ACVIM classification [1].

	Asymptomatic			Symptomatic	
Stages	A	B1	B2	C	D
Findings	Genetically predisposed breeds	- Murmur in the mitral valve - LA and LV dimensions are normal	- Murmur intensity ≥ 3/6 - VKS > 10.5 - LA/Ao ≥ 1.6 - LVIDd ≥ 1.7	- Tachypnea - Exercise intolerance - Cough - Dyspnea	- Tachypnea - Exercise intolerance - Cough - Dyspnea - Congestions
Drugs	None	None	Pimobendan or enalapril	Furosemide + enalapril or Pimobendan + enalapril	Furosemide + pimobendan + enalapril

**Table 2:** ICP-OES device parameters.

Parameters	Specified Values
Plasma gas flow rate	15 L/min
Argon flow rate	0.5 L/min
Sample flow rate	1.51 L/min
Peristaltic pump speed	100 rpm
RF Power	1150 W

**Table 3:** The results of the ICP-OES method validation for Cu, Zn, Fe, Co, Mg, Mn, Se, and Cr elements.

Elements	Quality Control (QC)	LOD (ppm)	LOQ (ppm)	Expected Concentration (ppm)	Measured Concentration (*n* = 3) (ppm)	Precision (RSD%)	Recovery (%)
Cu	QC-1 QC-2	0.003	0.008	0.500 1.000	0.536 0.963	0.752 0.213	107.2 96.3
Zn	QC-1 QC-2	0.004	0.006	0.500 1.000	0.464 1.035	0.712 0.394	92.8 103.5
Fe	QC-1 QC-2	0.004	0.008	0.500 1.000	0.509 0.990	0.321 0.675	101.8 99
Co	QC-1 QC-2 QC-3 QC-4	0.002	0.003	0.050 0.100 0.500 1.000	0.052 0.099 0.524 0.973	6.844 2.474 1.895 0.539	104 99 104.8 97.3
Mg	QC-1 QC-2	0.004	0.005	0.500 1.000	0.538 0.961	0.803 0.098	107.6 96.1
Mn	QC-1 QC-2	0.001	0.001	0.050 0.100	0.052 0.097	0.177 0.700	104 97
Se	QC-1 QC-2	0.002	0.003	0.050 0.100	0.049 0.100	0.726 0.816	98 100
Cr	QC-1 QC-2	0.000	0.001	0.050 0.100	0.052 0.097	4.503 1.740	104 97

QC: quality control; LOD: limit of detection; LOQ: limit of quantitation; RSD: relative standard deviation; ppm: mg/L.

**Table 4:** Wavelengths of each element in the ICP-OES measurements.

Element	Wavelength (nm)
Cu	324,754
Zn	206,200
Fe	259,940
Co	228,616
Mg	285,213
Mn	257,610
Se	196,090
Cr	267,716

**Table 5:** Serum Cu values (mg/L) according to treatment duration.

Treatment Groups	Min. 30 Days		Min. 120 Days		P ^1^
	Mean (SD)	Min.–Max.	Mean (SD)	Min.–Max.	
P	0.983 (0.695)	0.57–2.57	0.719 (0.143)	0.47–0.95	0.910
E	0.743 (0.088)	0.67–0.95	0.770 (0.535)	0.46–2.79	0.124
PE	0.728 (0.283)	0.42–1.44	0.613 (0.167)	0.35–0.95	0.346
EF	0.724 (0.378)	0.43–1.95	0.584 (0.069)	0.49–0.69	0.444
PEF	0.578 (0.188)	0.31–0.96	0.898 (0.763)	0.46–3.12	0.175
P ^2^	0.095		0.323		

^1^ Mann–Whitney U test significance level for the comparison of treatment duration for each treatment group; ^2^ Kruskal–Wallis test significance level for the comparison of different treatment groups for each treatment duration; P: pimobendan, E: enalapril, PE: pimobendan + enalapril, EF: enalapril + furosemide, PEF: pimobendan + enalapril + furosemide; mean (SD): arithmetic mean (standard deviation).

**Table 6:** Serum Zn values (mg/L) according to treatment duration.

Treatment Groups	Min. 30 Days		Min. 120 Days		P ^1^
	Mean (SD)	Min.–Max.	Mean (SD)	Min.–Max.	
P	0.683 (0.242)	0.37–1.10	0.668 (0.282)	0.23–1.10	0.910
E	0.704 (0.193)	0.49–1.08	0.663 (0.176)	0.34–1.05	0.798
PE	0.680 (0.225)	0.35–1.12	0.532 (0.160)	0.35–0.88	0.069
EF	0.621 (0.250)	0.22–1.06	0.851 (0.235)	0.59–1.14	0.051
PEF	0.608 (0.178)	0.30–0.90	0.772 (0.273)	0.33–1.28	0.152
P ^2^	0.890		0.051		

^1^ Mann–Whitney U test significance level for the comparison of treatment duration for each treatment group; ^2^ Kruskal–Wallis test significance level for the comparison of different treatment groups for each treatment duration; P: pimobendan, E: enalapril, PE: pimobendan + enalapril, EF: enalapril + furosemide, PEF: pimobendan + enalapril + furosemide; mean (SD): arithmetic mean (standard deviation).

**Table 7:** Serum Fe values (mg/L) according to treatment duration.

Treatment Groups	Min. 30 Days		Min. 120 Days		P ^1^
	Mean (SD)	Min.–Max.	Mean (SD)	Min.–Max.	
P	4.191 (3.428)	1.2–11.44	2.100 ^ab^ (0.419)	1.69–3.01	0.305
E	2.231 (0.568)	1.57–3.39	4.233 ^a^ (3.518)	0.29–14.43	0.066
PE	2.600 (1.009)	1.27–4.50	1.935 ^b^ (0.887)	0.74–3.76	0.123
EF	2.352 (0.902)	1.39–4.27	2.580 ^ab^ (0.745)	1.49–3.76	0.353
PEF	1.674 (0.722)	0.64–3.05	3.741 ^a^ (1.854)	1.69–7.74	0.002
P ^2^	0.055		0.012		

^1^ Mann–Whitney U test significance level for the comparison of treatment duration for each treatment group; ^2^ Kruskal–Wallis test significance level for the comparison of different treatment groups for each treatment duration; P: pimobendan, E: enalapril, PE: pimobendan + enalapril, EF: enalapril + furosemide, PEF: pimobendan + enalapril + furosemide; mean (SD): arithmetic mean (standard deviation). ^a,b^: Different letters in the same column indicate a *p* < 0.1 significance level.

**Table 8:** Serum Co values (mg/L) according to treatment duration.

Treatment Groups	Min. 30 Days		Min. 120 Days		P ^1^
	Mean (SD)	Min.–Max.	Mean (SD)	Min.–Max.	
P	0.019 (0.042)	0–0.15	0.033 (0.067)	0–0.20	0.27
E	0.009 (0.005)	0–0.20	0.106 (0.231)	0–0.88	0.798
PE	0.036 (0.105)	0–0.37	0.428 (1.340)	0–4.24	0.771
EF	0.004 (0.004)	0–0.01	0.004 (0.004)	0–0.10	0.968
PEF	0.006 (0.006)	0–0.02	0.124 (0.381)	0–1.28	0.261
P ^2^	0.630		0.381		

^1^ Mann–Whitney U test significance level for the comparison of treatment duration for each treatment group; ^2^ Kruskal–Wallis test significance level for the comparison of different treatment groups for each treatment duration; P: pimobendan, E: enalapril, PE: pimobendan + enalapril, EF: enalapril + furosemide, PEF: pimobendan + enalapril + furosemide; mean (SD): arithmetic mean (standard deviation).

**Table 9:** Serum Mg values (mg/L) according to treatment duration.

Treatment Groups	Min. 30 Days		Min. 120 Days		P ^1^
	Mean (SD)	Min.–Max.	Mean (SD)	Min.–Max.	
P	31.45 ^ab^ (18.50)	20.34–87.21	21.81 (2.581)	19.18–27.3	0.031
E	25.99 ^a^ (2.161)	23–30.2	26.57 (13.56)	12.64–77.01	0.124
PE	25.89 ^ab^ (15.33)	6.29–71.05	20.91 (1.645)	19.36–25.02	0.107
EF	27.90 ^ab^ (14.31)	20.46–75.52	24.45 (4.754)	19.53–31.08	0.602
PEF	21.32 ^b^ (2.284)	18.77–26.2	29.59 (16.32)	16.05–69.86	0.175
P ^2^	0.028		0.091		

^1^ Mann–Whitney U test significance level for the comparison of treatment duration for each treatment group; ^2^ Kruskal–Wallis test significance level for the comparison of different treatment groups for each treatment duration; P: pimobendan, E: enalapril, PE: pimobendan + enalapril, EF: enalapril + furosemide, PEF: pimobendan + enalapril + furosemide; mean (SD): arithmetic mean (standard deviation). ^a,b^: Different letters in the same column indicate a *p* < 0.05 significance level.

**Table 10:** Serum Mn values (mg/L) according to treatment duration.

Treatment Groups	Min. 30 Days		Min. 120 Days		P ^1^
	Mean (SD)	Min.–Max.	Mean (SD)	Min.–Max.	
P	0.006 (0.005)	0–0.02	0.004 (0.006)	0–0.02	0.473
E	0.004 (0.001)	0–0.01	0.004 (0.003)	0–0.01	0.315
PE	0.006 (0.004)	0–0.01	0.005 (0.004)	0–0.01	0.539
EF	0.006 (0.006)	0–0.02	0.005 (0.003)	0–0.01	0.904
PEF	0.007 (0.005)	0–0.01	0.006 (0.006)	0–0.02	0.656
P ^2^	0.939			0.598	

^1^ Mann–Whitney U test significance level for the comparison of treatment duration for each treatment group; ^2^ Kruskal–Wallis test significance level for the comparison of different treatment groups for each treatment duration; P: pimobendan, E: enalapril, PE: pimobendan + enalapril, EF: enalapril + furosemide, PEF: pimobendan + enalapril + furosemide; mean (SD): arithmetic mean (standard deviation).

**Table 11:** Serum Se values (mg/L) according to treatment duration.

Treatment Groups	Min. 30 Days		Min. 120 Days		P ^1^
	Mean (SD)	Min.–Max.	Mean (SD)	Min.–Max.	
P	0.523 (0.046)	0.20–0.83	0.404 (0.054)	0.18–0.60	0.115
E	0.527 (0.038)	0.42–0.74	0.489 (0.033)	0.23–0.80	0.504
PE	0.456 (0.048)	0.19–0.82	0.380 (0.030)	0.27–0.58	0.218
EF	0.449 (0.036)	0.24–0.67	0.447 (0.076)	0.14–0.66	0.977
PEF	0.425 (0.067)	0.17–0.75	0.577 (0.063)	0.18–0.88	0.119
P ^2^	0.491		0.055		

^1^ One-way analysis of variance significance level for the comparison of treatment durations for each treatment group; ^2^ independent samples *t*-test; P: pimobendan, E: enalapril, PE: pimobendan + enalapril, EF: enalapril + furosemide, PEF: pimobendan + enalapril + furosemide; mean (SD): arithmetic mean (standard deviation).

**Table 12:** Serum Cr values (mg/L) according to treatment duration.

Treatment Groups	Min. 30 Days		Min. 120 Days		P ^1^
	Mean (SD)	Min.–Max.	Mean (SD)	Min.–Max.	
P	0.012 (0.007)	0–0.03	0.012 (0.015)	0–0.05	0.734
E	0.006 (0.004)	0–0.01	0.011 (0.012)	1–0.05	0.175
PE	0.010 (0.007)	0–0.03	0.008 (0.005)	0–0.02	0.497
EF	0.011 (0.013)	0–0.05	0.013 (0.008)	0–0.03	0.353
PEF	0.006 (0.005)	0–0.02	0.008 (0.005)	0–0.02	0.710
P ^2^	0.337		0.763		

^1^ Mann–Whitney U test significance level for the comparison of treatment duration for each treatment group; ^2^ Kruskal–Wallis test significance level for the comparison of different treatment groups for each treatment duration; P: pimobendan, E: enalapril, PE: pimobendan + enalapril, EF: enalapril + furosemide, PEF: pimobendan + enalapril + furosemide; mean (SD): arithmetic mean (standard deviation).

## Data Availability

The original contributions presented in this study are included in the article. Further inquiries can be directed to the corresponding author.
